# 

**Published:** 1880-09

**Authors:** 


HERMAN MYNTER, M. D.,

S.G.O. INDEXED

### CONTENTS:

PAGE.

ORIGINAL COMMUNICATIONS:

Treatment of Strictures of the Nasal Duct, by Lucien Howe, M. D., . . . . 49
Recent Studies in Albuminuria, by H. S. Kilbourne, M. D., . . . . . . 54

CLINICAL REPORTS:

Fracture of the Acetabulum.—Autopsy. Reported by Dr C. A. McBeth, Service of Dr. C. C. F. Gay, . . . . . 60
Dislocation of the Hip into the Foramen Thyroideum—Reduction at the end of six weeks, by Herman Mynter, M. D., . 61

TRANSLATIONS:

Empyema as a Result of Pleuro-Pneumonia, from the Danish by Frederick Peterson, M. D., . . . . . . . . 64

SELECTIONS:

Professor Heidenhain's Views of Mesmerism as Reported by Professor Senator, at the Medical Society at Berlin, . . 66
Statistics of 250 cases of Cancer of the Breast, . . . . . . . . 69

PAGE.

Case of Aneurism of the Innominate Artery, presented by Dr. L. A. Stimpson, at the New York Surgical Society, . . . 71
Valvular Lesions of the Heart, . . . 72
Fatal Herniotomy Due to Abnormal Obturator Arteries, . . . . . . . 73
Hyoscyamia in Insanity, . . . . 75
The Substitution of a Lead Plate for a Portion of the Frontal Bone, by M. H. Post, M. D., St. Louis, . . . . . . 77
Thomas Keith and Ovariotomy. . . . 79
Cases of Neuralgia Treated with Tonga, by W. J. H. Lush, M. D., . . . . . 81
Crude Petroleum in Asthma, . . . . 83
Laceration of the Cervic Uteri as a Cause of Post-Partum Hemorrhage, . . . 84
The Treatment of Whooping-Cough with Atropia used Hypodermically and Carbolic Acid Inhalations, by Wm. Lee, M. D., . . . . . . . . 85
Second Attack of Constitutional Syphilis, . 87
Spencer Wells, . . . . . . . 89

EDITORIAL:

The Recent Medical Legislation, . . . 90
In Memoriam. . . . . . . . 92

REVIEWS, . . . . . . . 94

